# Marine Antimicrobial Peptides: Emerging Strategies Against Multidrug-Resistant and Biofilm-Forming Bacteria

**DOI:** 10.3390/antibiotics14080808

**Published:** 2025-08-07

**Authors:** Rita Magalhães, Dalila Mil-Homens, Sónia Cruz, Manuela Oliveira

**Affiliations:** 1 ECOMARE—Laboratory for Innovation and Sustainability of Marine Biological Resources, CESAM—Centre for Environmental and Marine Studies, Department of Biology, University of Aveiro, 3810-193 Aveiro, Portugal; sonia.cruz@ua.pt; 2 ITQB NOVA—Instituto de Tecnologia Química e Biológica António Xavier, NOVA University of Lisbon, Av. da República, 2780-157 Oeiras, Portugal; dalila.milhomens@itqb.unl.pt; 3 CIISA—Center for Interdisciplinary Research in Animal Health, Faculty of Veterinary Medicine, University of Lisbon, Av. Universidade Técnica, 1300-477 Lisbon, Portugal; 4 AL4AnimalS—Associate Laboratory for Animal and Veterinary Sciences, Faculty of Veterinary Medicine, University of Lisbon, Av. Universidade Técnica, 1300-477 Lisbon, Portugal; 5 cE3c—Centre for Ecology, Evolution and Environmental Changes, CHANGE—Global Change and Sustainability Institute, Faculty of Sciences, University of Lisbon, Campo Grande, 1749-016 Lisbon, Portugal

**Keywords:** antimicrobial peptides, biofilm, AMPs, antibiotic resistance, antibiotic tolerance, persister cells, marine

## Abstract

The global rise in antimicrobial resistance poses a major threat to public health, with multidrug-resistant bacterial infections expected to surpass cancer in mortality by 2050. As traditional antibiotic pipelines stagnate, novel therapeutic alternatives are critically needed. Antimicrobial peptides (AMPs), particularly those derived from marine organisms, have emerged as promising antimicrobial candidates due to their broad-spectrum activity, structural diversity, and distinctive mechanisms of action. Unlike conventional antibiotics, AMPs can disrupt microbial membranes, inhibit biofilm formation, and even modulate immune responses, making them highly effective against resistant bacteria. This review highlights the potential of marine AMPs as next-generation therapeutics, emphasizing their efficacy against multidrug-resistant pathogens and biofilm-associated infections. Furthermore, marine AMPs show promise in combating persister cells and disrupting quorum sensing pathways, offering new strategies for tackling chronic infections. Despite their potential, challenges such as production scalability and limited clinical validation remain; nevertheless, the use of new technologies and bioinformatic tools is accelerating the discovery and optimization of these peptides, paving the way for bypassing these challenges. This review consolidates current findings on marine AMPs, advocating for their continued exploration as viable tools in the fight against antimicrobial resistance.

## 1. Introduction

In recent decades, the world has faced a growing crisis due to antimicrobial resistance, with infections by resistant strains having directly and indirectly resulted in 4.71 million deaths in 2021 [1,2,3]. As this problem expands each year, mainly due to the overuse and misuse of antibiotics worldwide, infections caused by multidrug-resistant (MDR) bacteria are expected to become deadlier than cancer by 2050 [4]. Adding to the challenge, the development of new antibiotics has significantly slowed [5]. Therefore, it is mandatory to invest in the development of new drugs and alternatives for fighting resistant strains, to avoid the risk of returning to a pre-antibiotic era.

**Figure 1:**
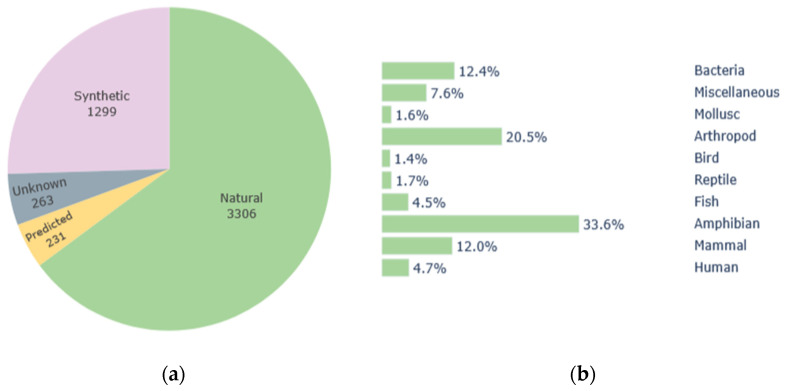
Antimicrobial peptides (AMPs) included in the Antimicrobial Peptide Database 3 (APD3) as of December 2024. (**a**) Distribution of the 5099 peptides according to their synthesis mechanism; (**b**) Distribution of AMPs according to different natural sources [10].

One alternative to conventional antibiotics that has attracted increasing attention from the scientific community for some years now is the use of antimicrobial peptides (AMPs) produced by plants and animals. AMPs are part of the innate immune system of all life forms and have shown a wide range of biological properties, such as antibacterial, antiviral, antiprotozoal, antifungal, anticancer, antioxidant, antihypertensive, antidiabetic, anticoagulant, anti-inflammatory, cardioprotective, neuroprotective and immunoregulatory effects [6,7,8]. As of December 2024, the Antimicrobial Peptide Database 3 (APD3) contains information on 5099 peptides, from which 3306 correspond to natural compounds produced by members of the six life kingdoms (Figure 1) [9,10,11,12,13,14,15]. This database includes 4648 entries corresponding to molecules with antibacterial potential, from which 172 show antibiofilm properties and 605 present activity against methicillin-resistant *Staphylococcus aureus* (MRSA) [10].

AMPs were first identified in the 1980s, with the first peptide from marine sources, tachyplesin, being described in 1988 [16]. Despite being discovered almost at the same time period, marine AMPs have received significantly less attention than their terrestrial counterparts, accounting for only ~4% of known AMPs in existing databases [17,18,19]. Seawater harbors greater biological and genetic diversity than any other environment, encompassing organisms from bacteria and small invertebrates to complex vertebrates, many of which exhibit unique adaptations to extreme conditions [17]. As such, it is expected that the number and diversity of bioactive molecules from marine organisms will also surpass those discovered in terrestrial lifeforms [19]. Despite this remarkable biodiversity, the exploration of the antibacterial potential of marine species continues to face numerous challenges, leaving much of its potential unexploited [17,20].

In recent years, the scientific community has developed and significantly improved Omics methodologies (genomics, transcriptomics, proteomics, metabolomics, and multiomics) and bioinformatic tools, which have played a pivotal role in the discovery of novel bioactive compounds, contributing significantly to the advancement of the field. These approaches enable high-throughput screening of marine genomes and transcriptomes, accelerating the discovery of peptides with antimicrobial potential [5,17].

In this review, marine AMPs are presented as alternatives to both conventional antibiotics and terrestrial AMPs in the fight against pathogenic MDR bacteria, including biofilm-forming strains. We first describe the general structural and functional characteristics of antimicrobial peptides, with an emphasis on marine-derived AMPs. We then explore their activity against multidrug-resistant bacteria, including their ability to inhibit biofilm formation and target persister cells. Finally, we discuss current limitations in AMP development and propose future directions for their clinical application. While the biological and antimicrobial properties of marine AMPs have been addressed in previous reviews, this work focuses on their antibacterial efficacy against both resistant and tolerant phenotypes.

## 2. Antimicrobial Peptides

AMPs are peptides with approximately 2 to 60 amino acids, and although some anionic AMPs have been described, generally they are positively charged, with +2 to +9 net charge. These molecules are expressed as part of the innate immune system of many organisms, being produced without eliciting harmful effects on the host organism [21,22]. Structurally, they are amphipathic, which enables them to easily attach to the membranes of the target pathogens, are cysteine-rich and present multiple intramolecular disulfide bridges [5,9,21,23,24]. Due to their broader range of biological effects in comparison with conventional antibiotics, AMPs are promising candidates for pharmacological applications [23]. Their wide spectrum of action, but also their toxicity, can be related to their structural diversity, which ranges from alpha helix to beta stand conformations [25,26,27].

**Figure 2:**
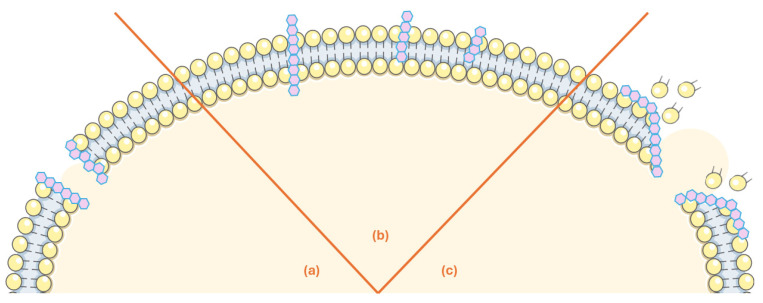
Effect of membrane-acting marine-derived antimicrobial peptides (AMPs). (a) Toroidal Pore Model: AMPs gather around the membrane forming a ring-shaped hole; (b) Barrel-Stave Model: AMPs’ multimers are able to penetrate the bacterial cell and establish channels that compromise membrane integrity and can also trigger apoptosis; (c) Carpet-like Model: AMPs act along the membrane surface to dissolve the phospholipidic bilayer.

AMPs are usually classified into five families according to their structure: linear α-helix peptides, β-sheet peptides, peptides with both an α-helix and β-sheet, peptides without an α-helix or β-sheet and topologically complex AMPs [28]. On another end, and even though AMPs present a great diversity of antimicrobial approaches, these peptides can be classified into two main categories according to their action mechanism: membrane-acting and non-membrane-acting [24]. In Figure 2, we represent the three main modes of action for membrane-acting peptides, namely the toroidal, barrel-stave and carpet-like models [24]. Their cationic nature is essential for the membrane-targeting mechanism, as it facilitates binding to the negatively charged phospholipidic membranes of pathogens. This, together with their ability to acquire amphipathic conformations, prompts the formation of pores in the microbial membranes, disrupting their integrity and promoting intracellular ion and metabolite leakage, which can culminate in cell death [26,29,30,31,32] (Figure 2).

AMPs exploit the differences between bacterial and eukaryotic membranes, which results in target selectivity. When targeting the membrane, AMPs link with the bilayer through their α-helix domains, while the β-folded domains are responsible for molecule stability and membrane crossing [33]. Besides acting at the membrane level, AMPs can also inhibit protein synthesis, negatively affect essential cellular processes and act as signalling molecules, modulating the host immune response [6,17]. Moreover, as they mainly act at the membrane level, their action mechanism limits the development of resistance, as this would require fundamental alterations of the bacterial membrane [34]. The reduced capacity of bacteria for developing resistance to AMPs, allied to their broad spectrum of activity, make these peptides perfect candidates for novel drug discovery [26].

## 3. Marine AMPs Against Antibiotic Resistance

Among the several sources of AMPs, marine species produce compounds with distinct biochemical properties and high therapeutic potential. Generally, marine creatures lack a developed acquired immune system, relying instead on innate immunity and defence strategies, in which AMPs are crucial [19,21,35]. When pathogen-associated molecular patterns (PAMPs) are detected in the surface of innate immunity receptors, signalling pathways are triggered, and phagocytosis and the production of antimicrobial substances are activated [36]. Most marine AMPs tolerate high salinity, pressure, and drastic fluctuations in pH, nutrient availability, oxygen levels and temperature, reflecting the highly competitive and demanding nature of their environment, contrary to their terrestrial and freshwater counterparts [1,12,34,37,38]. Likewise, organisms from the deep sea produce AMPs that are potentially better adapted to high-pressure environments, while AMPs from intertidal species tend to exhibit higher salt tolerance and resistance to desiccation [39]. In addition, marine AMPs usually present low cytotoxicity, high bioavailability and no haemolytic properties, although more in vivo studies are needed to confirm these traits [12,40]. These characteristics, combined with their high stability, support the previously proposed hypothesis that marine AMPs are ideal candidates for the development of next-generation antimicrobials.

Antimicrobial resistance can be inherent (intrinsic), acquired or a combination of both. Intrinsic antimicrobial resistance is a natural, universal trait of a bacterial species, making it innately non-susceptible to certain antibiotics, even without prior exposure to antimicrobials or the acquisition of resistance genes. Acquired resistance arises when previously susceptible bacteria develop resistance through genetic mutations or by acquiring resistance genes via horizontal gene transfer from other organisms. Resistance can occur through the production of antibiotic-inactivating enzymes, the modification of drug target sites, alterations in cell wall or outer membrane permeability, and the activation of antibiotic efflux pumps [4,41]. Many nosocomial infections are nowadays related to highly resistant bacteria, such as methicillin-resistant *Staphylococcus aureus* (MRSA), vancomycin-resistant enterococci (VRE), and Extended-spectrum beta-lactamase (ESBL) producing Gram-negative bacteria, resistant to all β-lactam antibiotics. The marine-derived AMPs that have demonstrated activity against multidrug-resistant bacteria are presented in Table 1.

## 4. Marine AMPs Against Biofilm

Beyond their activity against planktonic bacterial cells, marine AMPs have also shown significant potential in disrupting biofilms, the key bacterial structures responsible for chronic infections and consequently for increasing patients’ morbidity and mortality [62]. Biofilms are structured communities of microorganisms encased in a self-produced extracellular polymeric matrix, which attach to both living and non-living surfaces, such as medical devices, tissues, and industrial equipment. Biofilms can be composed of single or multiple microbial species, including bacteria and fungi, and are recognized for their ability to withstand external stressors—such as changes in temperature, pH, nutrient availability, and exposure to antimicrobial agents—far more effectively than their free-floating (planktonic) counterparts. This resilience is responsible for the persistence and chronicity of biofilm-associated infections, which account for 65 to 80% of human infections, especially those related to implanted devices and chronic wounds [63,64].

Biofilm communities confer several advantages to microorganisms, namely enhanced survival, metabolic adaptability, and protection from the action of both antibiotics and host immune responses [65,66,67,68]. In fact, bacteria within biofilms can exhibit antibiotic resistance levels exceeding 1000-fold compared to planktonic cells, largely due to the physical barrier of the extracellular matrix and the presence of metabolically dormant “persister” cells that evade conventional therapies without undergoing genetic changes. This makes the treatment of biofilm-related infections particularly challenging, especially of those associated with multidrug-resistant (MDR) pathogens such as *S. aureus*, *Pseudomonas aeruginosa*, and *Candida albicans* [69].

Given these challenges, there is a growing interest in alternative strategies able to disrupt biofilms, with AMPs from marine sources emerging as promising candidates. AMPs are multifaceted: they can rapidly kill cells at the early stages of biofilm formation, interfere with the extracellular matrix to hinder cell propagation, disrupt bacterial communication and quorum sensing (QS) systems, and can act synergistically with other antimicrobial drugs to enhance efficacy and reduce the risk of resistance development [24] (Table 2).

A particularly innovative approach to disrupting these bacterial communities involves targeting the QS systems that regulate biofilm formation and bacterial virulence. Quorum sensing inhibitors (QSIs) derived from marine microbes, such as cyclodipeptide diketopiperazines (DKPs), have been shown to impede QS-regulated pathogenicity, including biofilm development and the production of virulence factors. For example, cyclo (l-Trp-l-Ser) from the marine bacterium *Rheinheimera aquimaris* and cyclo (l-Tyr-l-Pro) from the fungus *Penicillium chrysogenum* were found to significantly inhibit biofilm formation and reduce virulence factor production by *P. aeruginosa*, a notorious biofilm-forming and MDR pathogen. Another marine-derived molecule, nesfactin, is able to inhibit biofilm formation by multidrug-resistant *P. aeruginosa* by 90% without affecting cell growth, being also able to degrade key QS signalling molecules [64,70,71].

Pleurocidin is a 25 amino-acid-long AMP derived from the winter flounder (*Pleuronectes americanus*), first described in 1997 [72]. Besides its antimicrobial activity against Gram-positive and Gram-negative bacteria, pleurocidin exhibits activity against drug-resistant *S. aureus* and can inhibit biofilm at 2 times its MIC (minimum inhibitory concentration). When compared with oxacillin and clindamycin, this AMP presented better results when eradicating bacterial biofilms at low concentrations [42]. A study investigating pleurocidin as a potential therapeutic for dental caries found out that, at a concentration of 64 µg/mL, this molecule is capable of reducing the *Streptococcus mutans* biofilm often associated with this condition by 75.2%, revealing its potential for use in formulations such as chewing gum or mouthwash [73].

An AMP named Tachyplesin III, consisting of 17 amino acids, is extracted from Southeast Asian horseshoe crabs and presents antibacterial, antifungal and antiviral activity. It has also been shown that ureteral stents impregnated with tachyplesin III can affect *P. aeruginosa* ATCC 27853 biofilms. When combined with the intraperitoneal administration of piperacillin and tazobactam, this treatment reduced bacterial counts to one quarter of the levels observed with either treatment alone in vivo [74].

Gaduscidin-1, an AMP derived from Atlantic cod (*Gadus morhua*), has emerged as a promising candidate for combating bacterial biofilm formation under cystic fibrosis conditions in vitro. When used in combination with conventional antibiotics, namely kanamycin and ciprofloxacin, Gad-1 enhanced their antibacterial efficacy. However, the cytotoxicity associated with Gad-1 is still a major challenge. To address this, researchers are currently developing a nanocapsule-based delivery system to mitigate Gad-1’s toxic effects and improve its therapeutic potential [75].

Reptiles have also been shown to produce AMPs with antibiofilm properties, as reported by Ouyang et al. in 2022 [76]. Cm-CATH2 from *Chelonia mydas* and Hc-CATH from *Hydrophis cyanocintus* revealed the ability to inhibit *Vibrio vulnificus* and *S. aureus* biofilms. Besides their permeabilization effect on the cytoplasmic membrane, these molecules were also able to modulate the immune response, inhibiting the expression of induced pro-inflammatory cytokines by bacteria while stimulating the phagocytic innate response of largemouth bass [76]. These AMPs possess strong activity against pathogenic bacteria in vivo, the ability to inhibit biofilm formation and a low propensity to induce resistance, while having multiple immunomodulatory effects [76,77,78].

In echinoderms, two AMPs from the sea-cucumber *Holothuria tubulosa* and one from the sea-urchin *Paracentrotus lividus* have been shown to inhibit biofilm formation by *S. aureus*, *S. epidermidis* and *P. aeruginosa*. These peptides only present modest activity against planktonic cells; however, their ability to inhibit biofilm formation is high, probably due to their specific mechanism of action [79,80]. AMPs produced by molluscs have also been shown to present antibiofilm properties [81,82].

Despite the extensive investigation of their antimicrobial properties, the antibiofilm potential of AMPs remains largely underexplored, which represents a promising opportunity for the future discovery of effective antimicrobial compounds. A list of natural-occurring AMPs from marine sources with antibiofilm activity can be found in Table 1.

It is important to note that, despite the advances in AMP research, no antibiofilm molecules have yet been approved for clinical use, so the discovery of new agents produced by marine microbial sources remains an active and promising area of research. The structural diversity and unique mechanisms of action of marine peptides offer significant potential for the development of novel therapeutic approaches capable of overcoming the defence mechanisms of biofilm-associated pathogens. As research continues, the integration of synthetic biology approaches may further enhance the efficacy and spectrum of these natural products, paving the way for the more effective management of biofilm-related infections [64].

Besides biofilms, persister cells also exhibit high tolerance to environmental stressors and resistance to antibiotic treatment, because these mainly target growth mechanisms and factors, which are inactive in persister cells. These cells are regulated by toxin–antitoxin systems, by alternative energy production mechanisms, by the SOS response to DNA damage, and by stringent responses, and can return to a normal growth rate once environmental conditions are ideal. Therefore, persistence is considered as adaptative resistance, which is associated with phenotypic variation [83,84,85,86]. Strategies to eradicate persister cells include the following: (a) the direct killing of the cells, even in their dormant state; (b) induction of resuscitation followed by conventional antibiotic therapy; (c) disruption of molecular pathways involved in persister cell formation to prevent their induction [87,88]. AMPs have been explored as a promising strategy to eliminate persister cells. Even though persisters can survive under stressful conditions, they still need an intact membrane to remain viable. Therefore, their ability to perturb the bacterial membrane make AMPs ideal candidates for the fight against persisters [89]. In the marine realm, Piscidine 3, an AMP from the hybrid striped seabass, was found to be effective against persisters due to its strong nuclease activity [90].

**Table 2:** List of AMPs from marine sources with antibiofilm properties. Abbreviations: MBIC, minimum biofilm inhibitory concentration; MIC, minimum inhibitory concentration; MBC, minimum bactericidal concentration; ROS, reactive oxygen species; QS, quorum sensing.

Compound	Source	Mechanism of Action	Antibiofilm Activity	References
Pleurocidin	Winter flounder (*Pleuronectes americanus*)	Membrane permeation and metabolic inhibition	*S treptococcus mutans* (75% reduction at 64 µg/mL); *S. aureus* (MBIC of 4 µM)	[42,45,73]
Tachyplesin III	Horseshoe crabs (*Tachypleus gigas* and *Carcinoscorpius rotundicauda*)	Disruption of the cell membrane structure and ROS production	*Pseudomonas aeruginosa* ATCC 27853 (MIC and MBC values of 4 and 32 µg/mL alone and 2 µg/mL and 8 µg/mL when associated with piperacillin-tazobactam)	[74,91]
Gaduscidin-1	Atlantic codfish (*Gadus morhua*)	Reducing biofilm adhesion and ROS production	*P. aeruginosa* PAO1 biofilms show less 15 to 27% adhesion values (0.5 μM)	[75]
Cm-CATH2	Green sea turtle (*Chlonia mydas*)	Membrane permeation and cell disruption	*Vibrio vulnificus* (73.68% reduction), *Staphylococcus aureus* CMCC26003 (77.77% reduction), *Enterococcus faecium* (93% reduction) and *S. aureus* (96% reduction)	[76,77]
Hc-CATH	Sea snake (*Hydrophis* *cyanocinctus*)	Membrane permeation and cell disruption	*V. vulnificus* (74.55% reduction) and *S. aureus* CMCC26003 (71.64% reduction)	[76]
Holothuroidin 1	Sea-cucumber (*Holothuria tubulosa)*	Interference with the initial bacterial adhesion, elimination of early bacterial colonizers and inhibition of QS	*S. aureus* ATCC 25923 (51.8% reduction at 3.2 3200 mg/mL; 37.9% reduction at 1500 3.2 mg/mL), *Staphylococcus epidermidis* ATCC 35984 (68.5% reduction at 3200 3.2 mg/mL; 58.2% reduction at 1500 3.2 mg/mL) and *P. aeruginosa* ATCC 15442 (69.9% reduction at 6200 3.2 mg/mL; 62.7% reduction at 3100 3.2 mg/mL)	[19,80]
Holothuroidin 2	Sea-cucumber (*Holothuria tubulosa)*	Interference with the initial bacterial adhesion, elimination of early bacterial colonizers and inhibition of QS	*S. aureus* ATCC 25923 (57.7% reduction at 3200 μg/mL; 40.5% reduction at 1500 μg/mL), *S. epidermidis* ATCC 35984 (73.8% reduction at 3200 μg/mL; 59.7% reduction at 1500 μg/mL) and *P. aeruginosa* ATCC 15442 (64.3% reduction at 6200 μg/mL; 43.8% reduction at 3100 μg/mL)	[19,80]
Paracentrin 1	Sea-urchin (*Paracentrotus lividus)*	Unknown	*S. aureus* 25923, *S. aureus* 29213, *S. aureus* 6538, *S. epidermidis* RP62A and *P. aeruginosa* 15442 (~80% at 6200 μg/mL)	[12,19,79]
Rpdef1α	Manila clam (*Ruditapes* *philippinarum*)	Reduction in the initial attachment or stimulation of bacteria motility	*Escherichia coli* MG1655	[82]
Phibilin	Two-striped slug *Philomycus bilineatus*	Prevention of the development of hyphae by destruction of the budding sites	Action against biofilm formation but also mature *Candida albicans* biofilms	[81]
Capitellacin	Polychaeta (*Capitella teleta*)	Membrane accumulation and consequent conductivity fluctuations. Membrane destruction when the threshold is reached	Action against biofilm formation but also *E scherichia coli* SBS 1936 mature biofilms	[92]
LFX01	*Lactiplantibacillus plantarum* strain LF-8	Unknown	*Shigella flexneri* 14	[93]
Pontifactin	*Pontibacter korlensis* SBK-47	Inhibition of microbial adhesion	*Bacillus subtilis* MRCC 619, *S. aureus* MTCC 96 and *Vibrio cholerae* MTCC3906 (99% reduction at 2000 μg/mL)	[94]
Pumilacidin-like cyclopeptide	*Bacillus* sp. 176	Inhibition of microbial motility and adhesion	*P. aeruginosa* and *B. subtilis* (>50% reduction at 300 μg/mL)	[95]
Cyclo (Trp-Ser)	*Rheinheimera aquimaris* QS102	Inhibition of QS	*P. aeruginosa* PAO1 (59.9% reduction at 200 μg/mL)	[70]
Cyclo (L-Trp-L-Pro)	*Penicillium chruso genum* DXY-1	Inhibition of QS	*P. aeruginosa* PAO1 (48% reduction at 500 μg/mL)	[71]
Nesfactin	*Nesterenkonia* sp. MSA31	Inhibition of QS	*P. aeruginosa* FSPA09 (90% reduction at 75,000 μg/mL)	[96]
Epicotripeptin	*Epicoccum nigrum* M13	Unknown	*B. subtilis* ATCC6633, *S. aureus* NRRLB-767 (100 µg/mL)	[97]
Crustin (Pp-Cru)	Blue swimmer crab *(Portunus pelagicus)*	Membrane permeation and cell disruption	*S. aureus, E. faecalis, P. aeruginosa, E. coli* (100 μg/mL)	[98]
Crustin (Ps-cr)	Green tiger shrimp *(Peaneaus semisulcatus)*	Inhibition of bacterial adherence	*Bacillus thuringiensis, B. pumilis, V. parahaemolyticus,* and *V. alginolyticuls* (40 μg/mL)	[99]

## 5. Current Challenges

Marine-derived AMPs have emerged as promising candidates due to their broad-spectrum activity and unique mechanisms of action, which make resistance development less likely. However, before AMPs can be considered a definitive solution to antimicrobial resistance, several challenges remain. Despite the plethora of information on their in vitro activities, significant gaps persist regarding their clinical applicability. Only a limited number of natural AMPs have even advanced to clinical trials, with the majority of research still confined to the preclinical stage [15,41]. Although the mechanisms of action of many marine AMPs have been partially elucidated, significant knowledge gaps remain, particularly regarding their pharmacokinetic profiles. In vivo, these peptides often present limited stability due to rapid enzymatic degradation and poor bioavailability caused by low permeability of nonbacterial membranes and rapid clearance from systemic circulation [100,101]. Moreover, in many cases, conventional antibiotics still show more efficacy than AMP applied alone. To enhance AMPs’ bioavailability and avoid these issues, some strategies are already in development (Table 3).

Finally, several challenges remain, including high production costs and technical limitations [110,111]. Chemical synthesis, while precise, is often costly and inefficient for large-scale production. Natural extraction from marine organisms is limited by low yields and the need for sustainable aquaculture systems. Semi-synthetic strategies and peptide engineering hold promise to overcome these hurdles but require further optimization and validation for clinical use. Continued innovation in scalable, cost-effective, and biologically compatible production methods is essential to translate marine AMPs into viable therapeutic agents [12,100,112].

## 6. Conclusions

Marine antimicrobial peptides (AMPs) represent promising candidates for the development of novel therapeutics to address antimicrobial resistance. Their structural diversity and broad-spectrum activity, including efficacy against multidrug-resistant pathogens, biofilm structures, and persister cells, make them valuable alternatives to conventional antibiotics. This review consolidates current findings on the mechanisms of action of marine AMPs and highlights key examples with proven in vitro and in vivo effectiveness. Despite these advances, their clinical application remains limited due to several challenges. Emerging technologies and continued research in this field are essential to fully realize the therapeutic potential of marine AMPs.

## 7. Future Directions

As Thomas and Antony stated, ‘the distinctiveness of marine antimicrobial peptides lies in their broad spectrum activity, mechanism of action, less cytotoxicity, and high stability, which form the benchmark for developing a potential therapeutic’ [12]. Fortunately, most challenges previously mentioned are likely to be solved as our understanding of these compounds continues to advance [21].

Synthetic or analog versions of marine AMPs can be developed with improved properties using biotechnological approaches, such as genetic engineering and Omic technologies, in order to optimize the production of pharmaceuticals from these sources [101,112,113,114]. Advances in bioinformatics and machine learning now enable the prediction of AMPs structure, function, and antimicrobial spectrum, accelerating the screening process and reducing experimental costs. Moreover, synthetic biology and peptidomimetic design allow the modification of marine peptides, improving stability, selectivity, and production scalability [100,101,114]. As these tools continue to evolve, they will be instrumental in addressing the current limitations related to marine AMPs, while also expanding the therapeutic pipeline of these compounds.

Marine-derived peptide drugs have made significant progress in pharmaceutical development, with notable examples such as ziconotide and brentuximab vedotin receiving FDA approval for clinical use [7,100]. The current global clinical pipeline includes 23 marine-derived compounds in various stages of clinical development, including plitidepsin (Aplidin^®^), PM00104, Kahalalide F, Hemiasterlin, Spisulosine, Pseudopterosin A, Salinosporamide A, Tetrodotoxin, Conotoxin G, Bryostatin 1, Demochlorella^®^ and Plinabulin, reflecting the ongoing interest and investment in this field [115,116,117,118,119,120,121,122].

Notably, marine-derived pharmaceuticals include not only peptides and proteins but also other bioactive molecules with applications in oncology, and beyond [8]. In addition to their pharmaceutical uses, AMPs from marine sources are gaining attention as potential food preservatives due to their safety, digestibility, solubility, and effectiveness under acidic conditions, making them safer alternatives to conventional preservatives [8]. These advances emphasize the biotechnological and therapeutic potential of marine bioactive compounds, which span a diverse range of chemical structures and bioactivities. Given the rise in antimicrobial resistance genes and mechanisms, including biofilm formation and cellular dormancy, it is essential to continue exploring alternatives to conventional antibiotics and to further investigate the hidden arsenal of antimicrobial properties that these small peptides possess.

## 8. Materials and Methods

To explore the antibacterial potential of marine AMPs, approximately 150 articles were identified through searches on PubMed^®^. Keywords such as “marine”, “antimicrobial peptides”, “biofilm” and “antimicrobial resistance” were used. Articles written in English and published in peer-reviewed journals were included, without a restriction on the publication date, to ensure a comprehensive review. Studies were carefully screened for relevance to the topic. The Antimicrobial Peptide Database (APD3) was consulted to identify marine AMPs with antibiofilm and anti-MRSA properties. Graph generation was performed using Python (version 3.10).

## Figures and Tables

**Table 1:** AMPs from marine sources with activity against multidrug-resistant bacteria. Abbreviations: MBIC, minimum biofilm inhibitory concentration; MRSA, methicillin-resistant *Staphylococcus aureus*.

Compound	Source	Mechanism of Action	Antibacterial Activity	References
Pleurocidin	Winter flounder (*Pleuronectes* *americanus*)	Membrane disruption, alteration of bacterial metabolic pathways and interference with quorum sensing	Active against multi-drug-resistant *Enterococcus faecium*, *Escherichia coli*, *Pseudomonas aeruginosa*, *Klebsiella pneumoniae*, and *Acinetobacter baumannii* (MIC values of 8–256 μg/mL)	[42,43,44,45,46,47]
Clavanins	Leathery sea squirt (*Styelaclava)*	When paired with Zn^2+^ ions there is an increased positive charge and membrane affinity, and therefore in membrane disruption ability	Active against MRSA ATCC 43300; MIC values of 16 μg/mL for clavanin C and 64 μg/mL for clavanin E	[48,49,50,51,52]
		Clavanin D and Clavaspirin can translocate inside the cells without damaging the membrane suggesting that they can interfere with processes inside the bacterial cell	Active against multi-drug-resistant *Enterobacter cloacae* when clavanin D is paired with clavaspirin	
Epinecidin-1	Grouper (*Epinephelus* *coioides*)	Membrane disruption and immunomodulation	Active against MRSA in mouse and pig models, *Helicobacter pylori*, and *P. aeruginosa*	[53,54,55,56,57]
Tilapia piscidin-3	Nile tilapia (*Oreochromis* *niloticus*)	Membrane disruption	Active against MRSA in mouse peritonitis models (≤40 μg/mL)	[58,59,60]
Tilapia piscidin-4	Nile tilapia (*Oreochromis* *niloticus*)	Membrane disruption and immunomodulation	Active against MRSA, carbapenem-resistant *A. baumannii* and resistant *K. pneumoniae* in vitro and MRSA in wound and peritonitis models (≤6.25 μg/mL)	[58,60,61]

**Table 3:** Potential strategies to bypass administration challenges of AMPs [35,43]. Abbreviations: AMP, Antimicrobial Peptide; NDEFgel, Nanodefensin-encased hydrogel.

Strategy	Mode of Action	Examples	References
AMP-antibiotic conjugates	Combination of antibiotics with membrane-interfering AMPs	Colistin and Bacteriocin Ampicilin and Arenin-I Vancomycin and LPS binding peptides	[34] [17,102,103,104] [105]
Nanonetworks	Net-like meshwork of fibrils that entangled the bacteria	Human α-defensin 6 ApBD1	[106] [107]
Nanoparticle-based drug delivery systems	Nanomaterials and hydrogels that can amplify the antibacterial spectrum and antimicrobial capacity of AMPs	Nanodefensin-encased hydrogel (NDEFgel)	[108,109]

## Data Availability

The datasets used and analyzed during the current study are available from the corresponding authors upon reasonable request.

## Abbreviations

The following abbreviations are used in this manuscript:

AMP	Antimicrobial Peptide
MDR	Multidrug Resistant
APD3	Antimicrobial Peptide Database 3
MRSA	Methicillin-resistant *Staphylococcus aureus*
MIC	Minimal Inhibitory Concentration
MBIC	Minimum Biofilm Inhibitory Concentration
ROS	Reactive Oxygen Species
VRE	Vancomycin-Resistant Enterococci
ESBL	Extended-Spectrum Beta-Lactamase
QS	Quorum Sensing
PAMPs	Pathogen-associated molecular patterns
QSI	Quorum sensing inhibitors
DKP	Diketopiperazine
